# Identification of Kinases Regulating Prostate Cancer Cell Growth Using an RNAi Phenotypic Screen

**DOI:** 10.1371/journal.pone.0038950

**Published:** 2012-06-27

**Authors:** Hilary Whitworth, Shriti Bhadel, Melissa Ivey, Mark Conaway, Andrea Spencer, Ronald Hernan, Heather Holemon, Daniel Gioeli

**Affiliations:** 1 Departments of Microbiology, Immunology, and Cancer Biology, University of Virginia, Charlottesville, Virginia, United States of America; 2 Department of Public Health Sciences, University of Virginia, Charlottesville, Virginia, United States of America; 3 Cancer Center Member, University of Virginia, Charlottesville, Virginia, United States of America; 4 Sigma-Aldrich Biotechnology, St. Louis, Missouri, United States of America; Northwestern University, United States of America

## Abstract

As prostate cancer progresses to castration-resistant disease, there is an increase in signal transduction activity. Most castration-resistant prostate tumors continue to express the androgen receptor (AR) as well as androgen-responsive genes, despite the near absence of circulating androgen in these patients. The AR is regulated not only by its cognate steroid hormone, but also by interactions with a constellation of co-regulatory and signaling molecules. Thus, the elevated signaling activity that occurs during progression to castration resistance can affect prostate cancer cell growth either through the AR or independent of the AR. In order to identify signaling pathways that regulate prostate cancer cell growth, we screened a panel of shRNAs targeting 673 human kinases against LNCaP prostate cancer cells grown in the presence and absence of hormone. The screen identified multiple shRNA clones against known and novel gene targets that regulate prostate cancer cell growth. Based on the magnitude of effect on growth, we selected six kinases for further study: MAP3K11, DGKD, ICK, CIT, GALK2, and PSKH1. Knockdown of these kinases decreased cell growth in both androgen-dependent and castration-resistant prostate cancer cells. However, these kinases had different effects on basal or androgen-induced transcriptional activity of AR target genes. MAP3K11 knockdown most consistently altered transcription of AR target genes, suggesting that MAP3K11 affected its growth inhibitory effect by modulating the AR transcriptional program. Consistent with MAP3K11 acting on the AR, knockdown of MAP3K11 inhibited AR Ser 650 phosphorylation, further supporting stress kinase regulation of AR phosphorylation. This study demonstrates the applicability of lentiviral-based shRNA for conducting phenotypic screens and identifies MAP3K11, DGKD, ICK, CIT, GALK2, and PSKH1 as regulators of prostate cancer cell growth. The thorough evaluation of these kinase targets will pave the way for developing more effective treatments for castration-resistant prostate cancer.

## Introduction

The androgen receptor (AR) is a critical regulator of prostate cancer progression and it is increasingly clear that the AR is regulated not only by its cognate steroid hormone, but also by interactions with a constellation of co-regulatory and signaling molecules [1]–[3]. For patients presenting with disseminated prostate cancer, the tumor is typically dependent on androgen for growth and therefore, initially responsive to surgical and/or pharmacological depletion of circulating androgens [4]. However, therapeutic success is temporary. The cancer almost invariably recurs and progresses to a metastatic and lethal disease. The extensive cross talk between signaling pathways, such as androgen and peptide signaling pathways, multiple genetic mutations, and the genetic plasticity of cancer, all contribute to the inherent and acquired resistance to androgen ablation [5].

Previous studies have demonstrated that polypeptide growth factor signal transduction pathways can stimulate AR activation, suggesting that the increase in growth factor and receptor expression could be causal in prostate cancer progression to castration resistance. Growth factor stimulation has been reported to render AR-responsive promoters hypersensitive to androgen [6]–[14], and forced over expression of HER2/neu in androgen-dependent prostate cancer cells has been shown to drive castration-resistant growth [15], [16]. Moreover, inhibition of EGFR/HER2 signaling can inhibit prostate cancer cell growth *in vitro* and *in vivo* [17], [18] as well as AR transcriptional activity, protein stability, DNA binding, and Ser 81 phosphorylation [19]. The ability of signaling cascades to influence AR function may play a significant role in the development and progression of prostate cancer where the increase in signal transduction activity has been associated with the acquisition of castration-resistant disease. This suggests that therapeutic strategies targeting kinase cascades can overcome the compensatory signaling mechanisms that limit the effectiveness of androgen ablation.

In order to identify the signaling pathways that regulate prostate cancer cell growth, we screened a panel of shRNAs that target the human kinome against LNCaP prostate cancer cells grown in the presence and absence of androgen. We searched for kinases that had general growth effects and kinases that compensated for androgen ablation. The screen identified multiple shRNA clones against gene targets that regulate both androgen sensitivity and cell growth. We report here the results of our screen and the detailed evaluation of a subset of kinases identified as regulators of prostate cancer cell growth.

## Results

**Figure 1:**
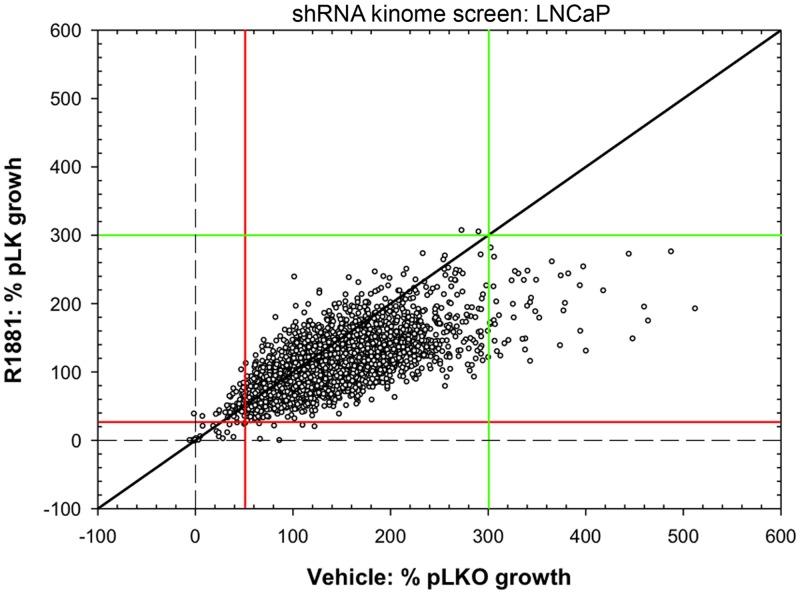
shRNA kinome-wide screen. LNCaP cells were transduced in triplicate with three to five shRNAs targeted against 673 human kinases. Cell growth was measured by alamarBlue on day 7. Plotted is the cell growth relative to pLKO empty vector control in response to each shRNA in the presence and absence of hormone (0.05 nM R1881). The red and green lines demarcate cut off points based on controls including pLKO, NTC, media alone, and AR shRNA. The red line indicates growth inhibition and the green line growth.

Prior to screening a panel of shRNAs that target the human kinome against LNCaP prostate cancer cells, we performed careful optimization of parameters, including cell growth conditions, multiplicity of infection, puromycin selection, androgen treatment, and cell viability measurements (data not shown). We identified shRNA clones that decreased and increased LNCaP cell growth in both the presence and absence of androgen (**Figure 1**). The screen used the MISSION® library with three to five shRNAs for each of 673 kinase targets; three independent biological replicates were performed on separate days. Following knockdown, alterations in LNCaP cell metabolism were determined using AlamarBlue as a surrogate for cell proliferation. Multiple shRNA clones against gene targets affecting cell growth were identified.

There was no difference in growth when comparing pLKO empty vector to a non-target control (NTC) (n = 82, data not shown). Androgen-treated cells grew 3.2 fold greater than vehicle-treated cells (n = 82). AR knockdown using shRNA was used as a positive control in the screen, inhibiting growth by more than 60% in cells grown in the presence of androgen (n = 41, data not shown) but having minimal effect on LNCaP cells grown in the absence of androgen (n = 41, data not shown). In the presence of androgen, we scored shRNAs that inhibited growth by at least 75%, which represents less than the top 1% of shRNAs inhibiting growth, as shRNAs targeting kinases that positively regulate LNCaP cell growth. In the absence of androgen, we scored shRNAs that inhibited growth by 50% or more, which represents less than the top 2% of shRNAs inhibiting growth, as shRNAs targeting kinases that positively regulate LNCaP cell growth. Using these criteria, shRNA knockdown of 46 kinases inhibited cell growth. Interestingly, very few shRNAs showed an effect that was dependent on the presence or absence of androgen. Most shRNAs inhibited growth under both conditions, although the magnitude of inhibition varied, indicating that this screen did not reveal kinases that specifically regulate androgen-induced LNCaP cell growth. Ribosomal protein S6 kinase (RPS6KA3), which has been implicated in regulating AR activity and prostate cancer cell growth [20]–[24], was identified in this screen, supporting an RNAi screening approach to identify kinases regulating prostate cancer cell growth. We also observed 34 kinases representing the top 1%, whose knockdown increased LNCaP cell growth (**Figure 1**).

We selected six inhibitory kinases for further study based on the magnitude of effect by shRNA knockdown: mitogen-activated protein kinase kinase kinase 11 (MAP3K11), diacylglycerol kinase delta (DGKD), intestinal cell kinase (ICK), citron rho interacting kinase (CIT), galactokinase2 (GALK2), and protein serine kinase H1 (PSKH1). One prediction posits that if the kinases that decrease growth when knocked down are causal in prostate cancer progression, then the activity of these kinases should increase during cancer progression. As an intermediate step to examining the activation state of the kinases, we examined kinase message levels in the Oncomine database. We found that in at least two independent studies the mRNA levels for the six kinases increased either when primary prostate cancer is compared to normal prostate or when metastatic prostate cancer is compared to primary disease or normal prostate (**Figure S1**).

**Figure 2:**
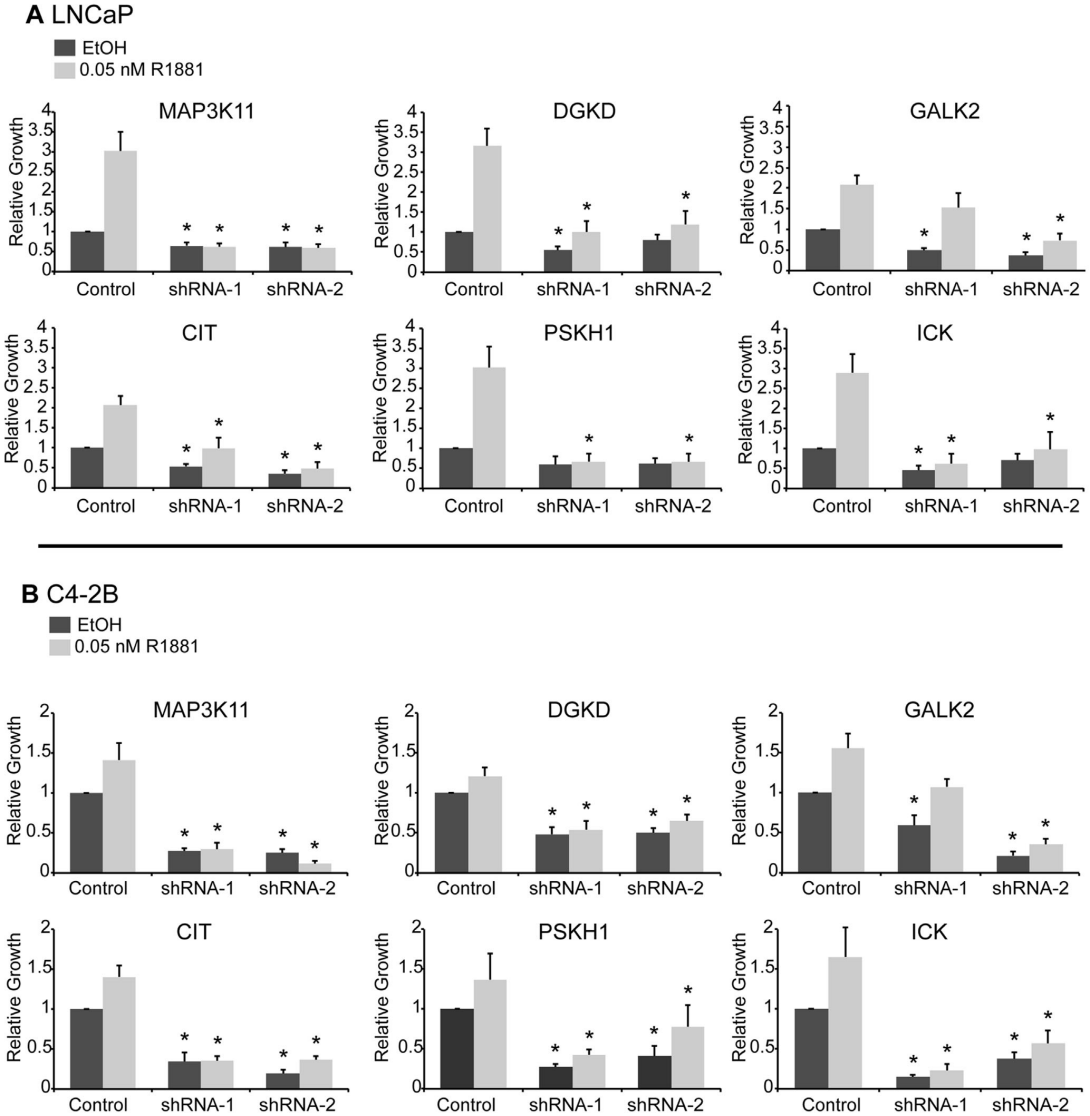
Kinase knockdown effect on growth. The relative effect of two independent shRNAs per kinase on cell growth in LNCaP (A) and C4-2B (B) cells. CyQuant Assay measured DNA content as a surrogate for cell number 7 days after shRNA transduction. The experiment was done in the presence and absence of hormone (0.05 nM R1881), n = 3 to 7 depending on the kinase. Cell growth was compared to untreated pLKO control and the values were averaged across biological replicates. Error bars represent standard error of the mean. ∗ denotes statistical significance.

We validated the growth effect and knockdown of our six selected kinases using the CyQuant Assay, which measures DNA content as a surrogate for cell number, and used this technique to also extend our analysis to the castration-resistant cell line, C4-2B. The cells were transduced with lentiviral particles expressing two shRNAs specific for each kinase of interest or pLKO empty vector control in the presence (0.05 nM R1881) or absence of androgen. As observed in Figure 2, growth was decreased in both cell lines in response to each shRNA. In general, kinase knockdown inhibited growth in the presence and absence of androgen. Furthermore, kinase knockdown affected growth equivalently in both the androgen-dependent LNCaP and castration-resistant C4-2B cell line.

**Figure 3:**
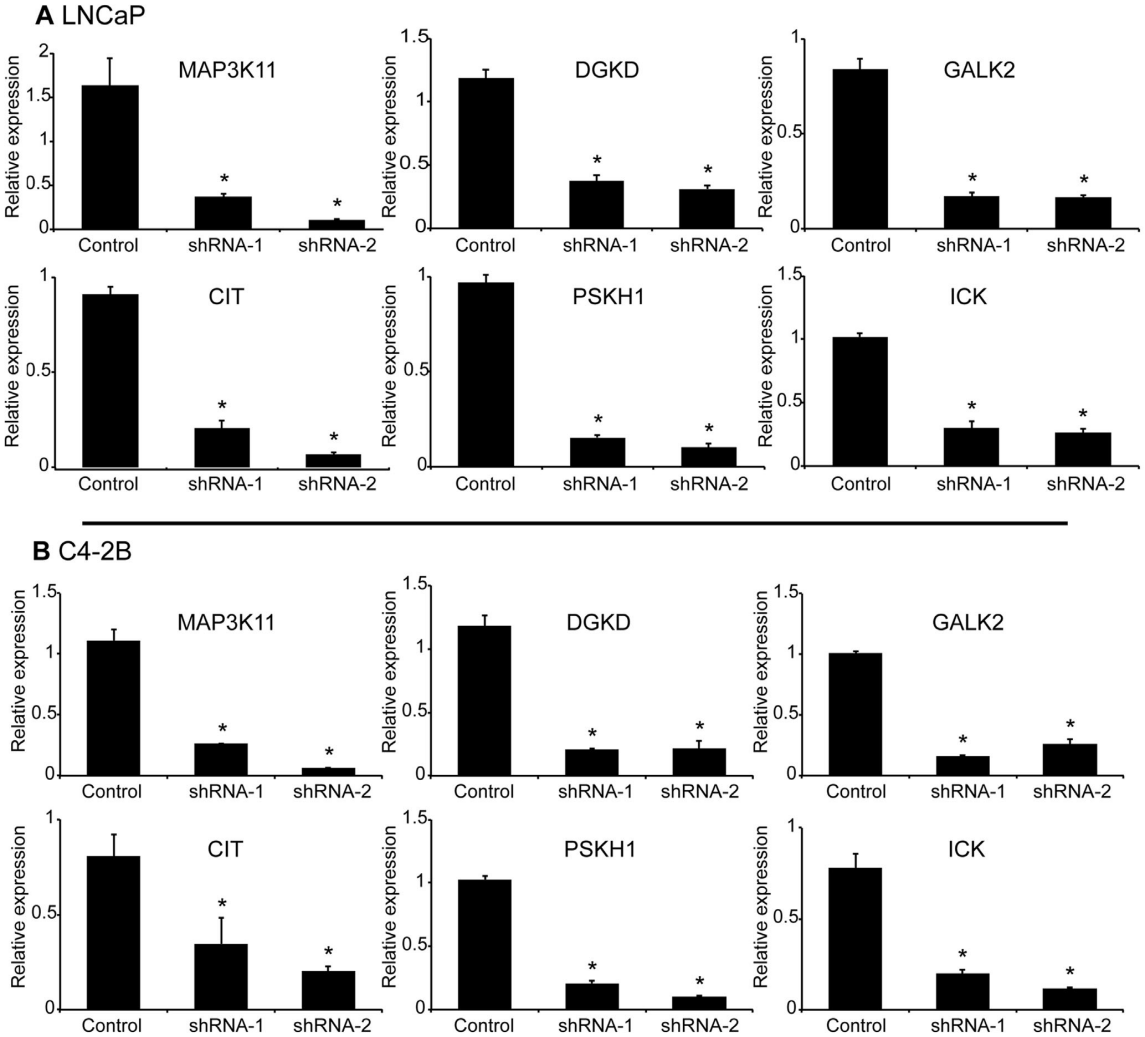
shRNA effect on target expression. Kinase transcript levels in LNCaP (A) and C4-2B (B) cells following knockdown with two independent shRNAs per kinase. Transcript levels were measured by qPCR on day 4 following transduction and 2 hours after R1881 hormone treatment (vehicle, 0.05, 0.5, and 1 nM). Transcript levels were compared to untreated pLKO control and normalized to the housekeeping gene, PSMB6. Values were averaged across hormone concentrations and biological replicates. Error bars represent standard error of the mean. ∗ denotes statistical significance.

qPCR was used to determine kinase knockdown by shRNA in LNCaP and C4-2B cells (**Figure 3**). Hormone was added at various concentrations (vehicle, 0.05, 0.5, and 1 nM R1881) and RNA was isolated at 2 and 24 hours following hormone treatment. These hormone treatments were the same as those used to assess the effect of kinase knockdown on AR transcriptional activity, which is described below and presented in Table 1. Each kinase was knocked down in both cell lines with two different shRNAs and compared to the pLKO empty vector control. We did not observe an effect of hormone dose on the efficiency of kinase knockdown (**Figure S2**); thus, the data shown in Figure 3 are the qPCR values averaged across biological replicates and hormone concentrations for each shRNA or the pLKO control at 24 hours following hormone stimulation. The shRNA viruses elicit greater than 50% knockdown of the target kinase mRNA as compared to pLKO, with most knockdowns greater than 70% at both time points and in both cell lines. Essentially identical observations were made for kinase knockdown following 2 hours of hormone stimulation (data not shown).

**Table 1:** AR transcriptional activity in response to kinase knockdown.

	LNCaP				C4-2B			
	TMPRSS2		SGK		TMPRSS2		SGK	
	2	24	2	24	2	24	2	24
CIT	−	↓	↑	−	−	−	↑	−
DGKD	−	−	−	↓	−	↓	−	−
GALK2	−	−	−	↑	−	−	↑	↑
ICK	−	−	−	−	−	−	−	−
PSKH1	−	−	−	−	−	−	−	−
MAP3K11	−	↓	−	−	↓	−	−	↑

Each of the six kinases was knocked down using two independent shRNAs in LNCaP and C42B cells. Cells were treated with varying levels of R1881 (0, 0.05 nM, 0.5 nM, 1 nM) for 2 or 24 hrs. Transcript levels of two AR target genes, TMPRSS2 and SGK, were measured by qPCR and compared to pLKO control and normalized to the housekeeping gene, PSMB6. Arrows represent statistically significant up or down regulation of mRNA levels.

There was some differential knockdown of kinase mRNA by shRNA, which may account for the differential knockdown of growth. For example, in LNCaP cells, CIT shRNA-2 elicited a greater growth inhibition than CIT shRNA-1, which parallels the effect on kinase mRNA knockdown, where CIT shRNA-2 reduced CIT mRNA levels more than CIT shRNA-1. However, the parallels between growth inhibition and mRNA knockdown are not evident for all kinases targeted.

**Figure 4:**
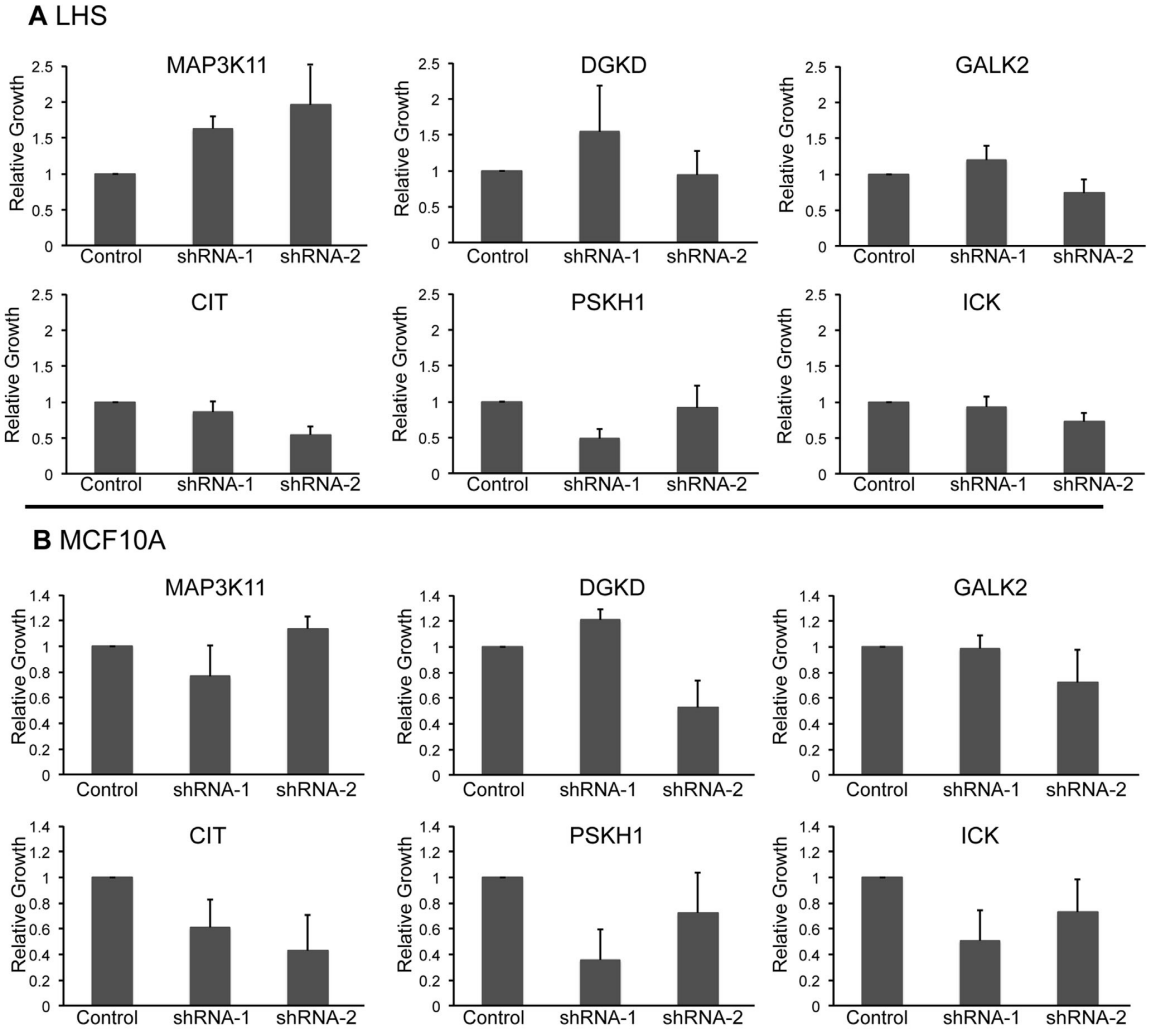
Kinase knockdown has minimal effect on non-cancer cells. The relative effect of two independent shRNAs per kinase on cell growth in LHS (A) and MCF10A (B) cells. CyQuant Assay measured DNA content as a surrogate for cell number 7 days after shRNA transduction. n = 2 for LHS cells and n = 3 for MCF10A cells. Cell growth was compared to pLKO control and the values were averaged across biological replicates. Error bars represent standard error of the mean.

In order to determine if the inhibition of growth induced by kinase knockdown was specific to prostate cancer cells, we measured the growth of LHS and MCF10A cells in response to shRNA targeting the seven kinases (**Figure 4**). LHS cells are non-tumorigenic immortalized human prostate epithelial cells generated by ectopic expression of SV40 large, small T antigen, and human telomerase [25]. MCF10A is a non-tumorigenic, spontaneously immortalized breast epithelial cell line [26]. LNCaP cells served as a control, with parallel experiments demonstrating inhibition of LNCaP cell growth and kinase expression (data not shown). In general, shRNA directed against the kinases had minimal effect on LHS and MCF10A cell growth, suggesting selectivity towards prostate cancer cells (**Figure 4**). The knockdown of kinase message in LHS and MCF10A cells was variable (data not shown). In LHS cells, MAP3K11 was effectively knocked down (75 to 90% inhibition) and PSKH1 (50% inhibition); however the knockdown was inefficient for the other kinases. In MCF10A cells, CIT was inhibited (80%) and PSKH1, DGKD, and GALK2 were each inhibited by approximately 50%. The inability to inhibit kinase expression to a similar extent as in LNCaP, C4-2B, (**Figure 3**) and CWR22Rv1 cells (data not shown), complicates interpreting the importance of these kinases in normal cell growth and survival. However, all six kinases were knocked down in at least one of the normal cell lines tested. Thus, these results are consistent with there being selectivity for targeting these kinases in cancer cells over normal cells.

Since the AR is a major regulator of prostate cancer cell growth, we wanted to determine if any of the six selected kinases might affect growth through regulating the AR transcriptome. To examine the effect of kinase knockdown on AR target gene transcription, qPCR was used to measure transcript levels of two AR target genes, TMPRSS2 and SGK, in LNCaP and C4-2B cells with two independent shRNAs used to inhibit kinase expression (**Table 1**). We examined transcription of these genes at 2 and 24 hours to evaluate the effect of kinase knockdown on the immediate-early response and steady-state levels of AR transcriptional activity. Statistical analysis indicates that there was no effect of hormone dose on the ability of kinase knockdown to affect AR transcription; kinase knockdown altered transcription equivalently, or had no effect, at each androgen dose. Maintenance of androgen induction in pLKO was observed in all analyzed experiments. Reported in Table 1 are the statistically significant changes in AR transcription of TMPRSS2 and SGK in response to kinase knockdown by two independent shRNAs at 2 and 24 hours post three different androgen dose treatments. Both shRNAs had to alter gene transcription significantly in the same direction for reporting in the table.

There was no consistent decrease in AR transcriptional activity in response to knockdown of the six kinases across both cell lines, AR target genes examined, and the two time points tested (**Table 1**). Knockdown of MAP3K11 decreased transcription of TMPRSS2 in LNCaP cells at 24 hours and in C4-2B cells at 2 hours after the addition of androgen. However, MAP3K11 knockdown increased transcription of SGK in C4-2B cells 2 hours after the addition or hormone. Knockdown of DGKD decreased transcription of TMPRSS2 in C4-2B cells and of SGK in LNCaP cells at 24 hours. There was no change in TMPRSS2 or SGK transcription in either cell line as a result of the knockdown of ICK or PSKH1. CIT knockdown has disparate effects on AR transcription. Interestingly, the most consistent effect on AR transcription was from GALK2 knockdown, which caused an increase in SGK at 24 hours after hormone addition in both cell lines and at 2 hours in C4-2B cells. However, examining only TMPRSS2 and SGK as representative AR target genes may create selection bias; therefore, we expanded our analysis of AR-regulated genes.

**Figure 5:**
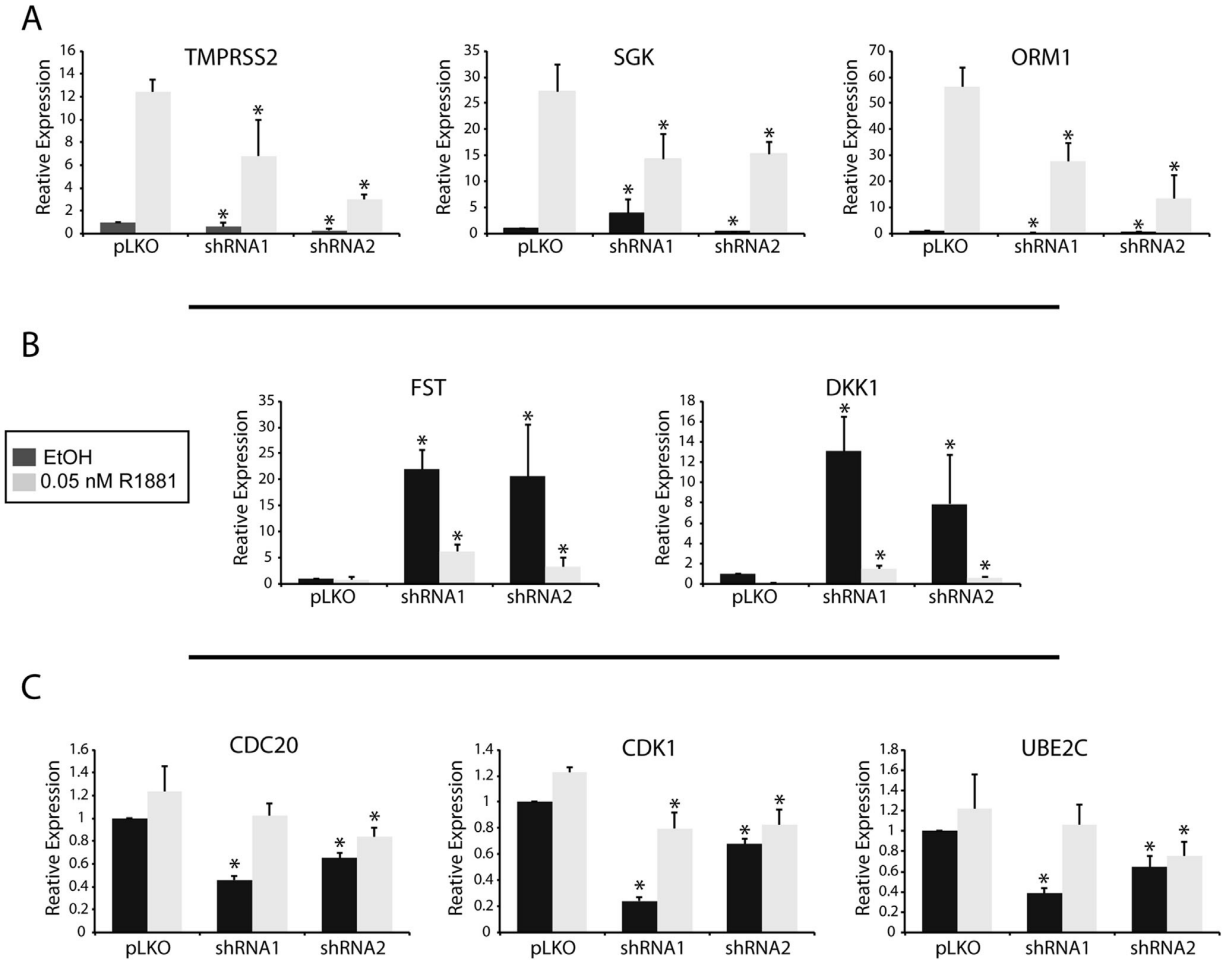
AR transcriptional activity in response to MAP3K11 knockdown. (A) Transcript levels of androgen-induced AR target genes that changed in response to MAP3K11 knockdown in LNCaP cells transduced with two independent shRNAs and pLKO control. RNA was isolated 24 hours after addition of R1881 at 1 nM. Transcript levels were measured by qPCR, compared to pLKO and normalized to the housekeeping gene, GUS. (B) Transcript levels of androgen-repressed AR target genes and (C) transcript levels of AR-regulated M-phase genes described in [27]. (B) and (C) were processed as described for (A). Values were averaged across biological replicates, n = 3. Error bars represent standard error of the mean. ∗ denotes statistical significance.

We analyzed 14 additional genes, including the AR-activated genes PSA, FKBP51, ORM1, STAG, Nkx3.1, FASN, AQP3, KLK2, and UGT2B; the AR-repressed genes DKK and FST; and the castration-resistant prostate cancer AR-regulated genes CDC20, CDK1, and UBE2C. Transcription in LNCaP cells following MAP3K11 knockdown was examined at 24 hours after androgen treatment. As expected, androgen induced or repressed transcription of all AR target genes in pLKO control cells. The effect of MAP3K11 knockdown on AR transcription is target dependent. Androgen-stimulated transcription of TMPRSS2, SGK, and ORM1 was reduced in response to MAP3K11 knockdown (**Figure 5A**). The inverse was true for the androgen-repressed genes DKK and FST. MAP3K11 knockdown stimulated gene expression and diminished the amount of androgen-induced repression (**Figure 5B**). PSA, FKBP51, STAG, Nkx3.1, FASN, AQP3, KLK2, and UGT2B did not change in response to MAP3K11 knockdown. Insofar as this subset of AR target genes represents the AR transcriptome, these data suggest that the inhibition of cell growth in response to MAP3K11 kinase knockdown may be due to regulation of AR transcriptional activity on a subset of AR-regulated genes. The AR selectively regulates cell cycle regulated genes such as CDC20, CDK1, and UBE2C to promote cell growth [27]. We found that MAP3K11 knockdown led to a slight, but statistically significant reduction in transcription of these M-phase AR-regulated genes (**Figure 5C**).

**Figure 6:**
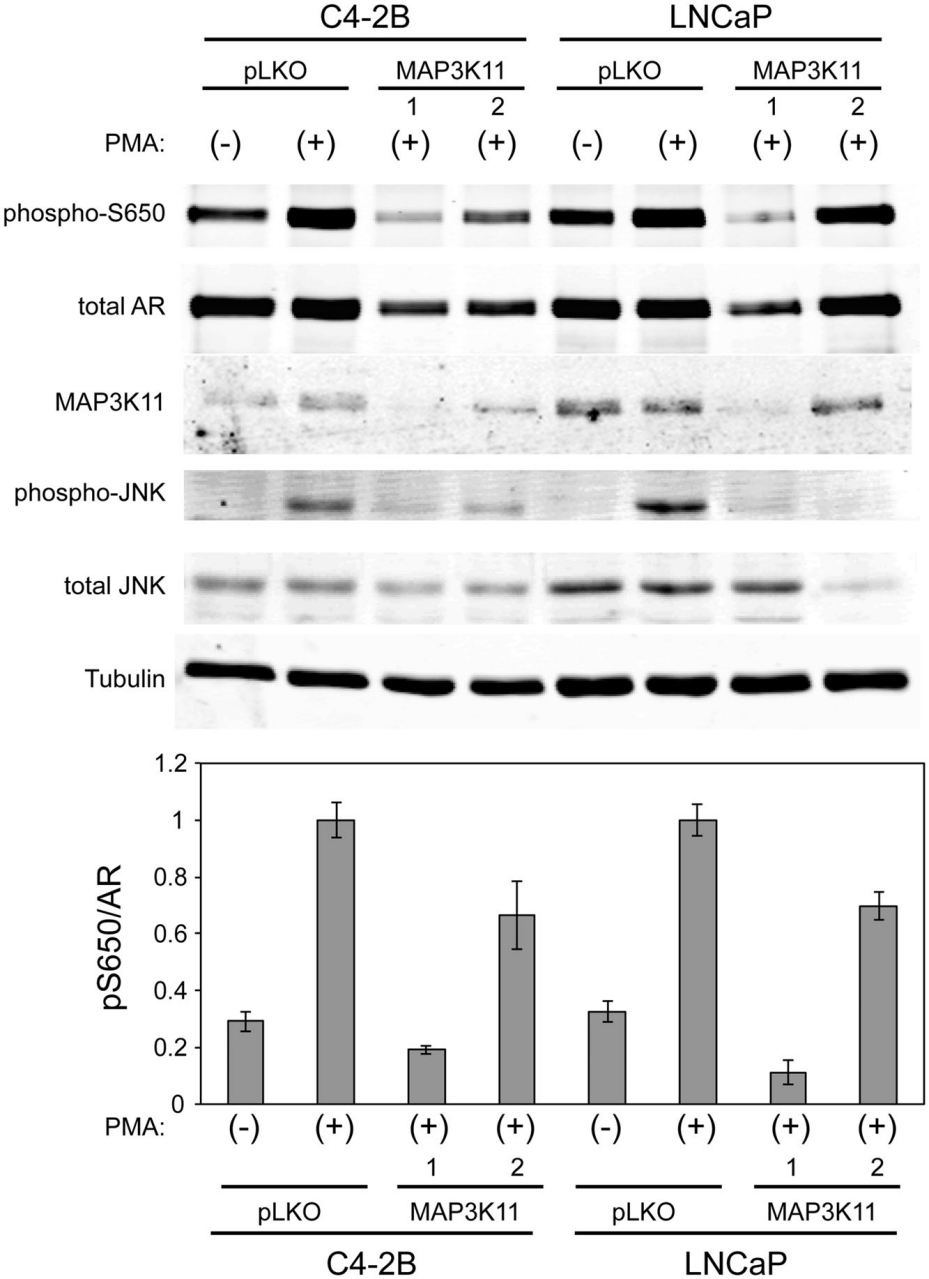
MAP3K11 regulates AR S650 phosphorylation. LNCaP and C4-2B cells were transduced with two independent shRNAs targeting MAP3K11 or pLKO control. Cells were treated with either vehicle or PMA. Total AR was immunoprecipitated and blotted for phospho-S650 and total AR levels. Cell lysate was blotted for total MAP3K11, total JNK, phospho-JNK, and tubulin. Plotted is the phospho-S650 signal normalized to total AR, n = 3. Quantitation was performed on Odyssey LICOR imaging system. Error bars represent standard error of the mean.

Previously, we demonstrated that stress kinases could regulate AR Ser 650 phosphorylation [28]. Thus, to further explore the mechanism of MAP3K11 regulation of prostate cancer cell growth and AR transcription, we tested if MAP3K11 knockdown regulated AR Ser 650 phosphorylation since MAP3K11 is an upstream regulator of JNK activity. PMA induced AR Ser 650 phosphorylation in LNCaP and C4-2B cells more than 3 fold (**Figure 6**). In these experiments, both shRNAs decreased MAP3K11 protein expression, with MAP3K11 shRNA-1 decreasing expression to a greater extent than MAP3K11 shRNA-2. Similar alterations in c-Jun phosphorylation were observed, suggesting that the MAP3K11 shRNAs inhibited PMA-induced stress kinase signaling. Under these conditions, AR Ser 650 phosphorylation was decreased in both LNCaP and C4-2B cells. A parallel decrease in total AR was observed. Quantitation of the relative amount of Ser 650 phosphorylation to total AR showed a reduction in phospho-Ser 650 (**Figure 6** histogram), suggesting a stoichiometric change in AR Ser 650 phosphorylation in response to MAPK3K11 knockdown. MAP3K11 shRNA-1 had the greatest impact on AR Ser 650 phosphorylation, paralleling the effect on MAP3K11 protein expression and c-Jun phosphorylation. These observations are consistent with our earlier results which suggest that stress kinase signaling regulates AR Ser 650 phosphorylation [28] and further suggest that MAP3K11 knockdown may be eliciting growth inhibition through interruption of the AR.

## Discussion

Our study demonstrates the applicability of lentiviral-based shRNA for conducting phenotypic screens to identify targets involved in prostate cancer growth. The signaling pathways regulating prostate cancer cell growth and AR activity play a pivotal role in the transition from androgen-dependent prostate cancer to castration-resistant disease [29]. In order to identify kinases within these networks, we examined how RNAi knockdown of kinases affects LNCaP prostate cancer cell growth. We predicted finding both novel and known regulators of growth. The screen identified kinases previously shown to regulate prostate cancer cell growth and AR activity, including the ribosomal S6 kinase (RPS6KA3 or RSK) [30]. RSK is downstream of MAPK and enhances PSA transcription. Chemical inhibition of RSK decreased PSA expression and prostate cancer cell growth [30]. RSK appears to mediate AR transcriptional activation through its own kinase function as well as interactions with p300, an AR co-regulator with HAT activity. Identifying RSK in our screen supports hypomorphic genetic screens to identify kinases regulating prostate cancer cell growth.

Multiple targets were identified in our RNAi screen, suggesting that multiple kinase signaling pathways regulate prostate cancer cell growth. We selected six kinases for further study, including MAP3K11, DGKD, ICK, CIT, GALK2, and PSKH1. Knockdown of all six kinases was found to decrease cell growth in both androgen-dependent and castration-resistant prostate cancer cells. To determine if the growth effect was mediated through the AR, we initially examined transcription of two well-characterized AR responsive genes, TMPRSS2 and SGK. We did not observe a consistent effect across the time points and cell lines tested on AR transcription of TMPRSS2 and SGK when the six kinases were knocked down. However, when we expanded our analysis to 16 total AR-regulated genes in response to MAP3K11 knockdown, we found that a subset of AR target genes was altered. This suggests that MAP3K11 knockdown can modulate AR transcriptional activity and may mediate its growth effects through the AR. Additionally, these data add to the evidence that the AR can be regulated in a promoter selective manner, allowing it to serve as an integrator of multiple extracellular signals. Our lab and others have reported this in previous studies [31], [32]. Further experiments involving additional AR target genes will be necessary to fully evaluate the effects of knockdown of these six kinases on AR transcription.

MAP3K11, also called Mixed Lineage Kinase (MLK3), is a member of the serine/threonine kinase family that preferentially activates MAPK8/JNK kinase and functions as a positive regulator of JNK signaling [33]. Additionally, this kinase can directly phosphorylate and activate IkappaB kinase and is involved in the transcriptional activity of NF-kappaB mediated by Rho family GTPases. MAP3K11 is required for serum-stimulated cell proliferation and for mitogen and cytokine activation of p38, ERK, and JNK1. MAP3K11 also plays a role in mitogen-stimulated phosphorylation and activation of BRAF, without phosphorylating BRAF directly. Thus, MAP3K11 functions as a node in the mitogen and stress signaling pathways. We have previously shown that activation of the MAP kinase pathway correlates with prostate cancer progression in a variety of settings and determined that stress kinase signaling regulates AR Ser 650 phosphorylation [28], [34]. In this study, we confirmed that stress kinase signaling regulates AR Ser 650 phosphorylation; knockdown of MAP3K11 stoichiometrically decreased PMA-induced AR Ser 650 phosphorylation. Modulation of Ser 650 phosphorylation may be regulating AR transcriptional activity of the AR target genes that were altered upon MAP3K11 knockdown, including TMPRSS2, SGK, ORM1, DKK and FST. We also found that the castration-resistant prostate cancer AR regulated M-phase genes CDC20, CDK1, and UBE2C [27], were decreased in response to MAP3K11 knockdown, although the decrease in transcription of these genes may reflect the inhibition of growth triggered by MAP3K11 knockdown and not represent altered AR transcriptional activity. Our screen also identified other stress kinases, including MAP3K7, MAP4K3, and MAPKAPK5 (data not shown), which underscores the critical nature of stress kinase signaling in regulating prostate cancer cell growth.

DGKD is an enzyme that phosphorylates diacylglycerol (DAG) to produce phosphatidic acid (PA). DGK catalyzes the phosphorylation of DAG by converting it to PA, thereby exchanging one second messenger for another and activating protein kinase C (PKC) [35]. There is increasing evidence suggesting that DGKD is involved in regulating DAG and PA levels in response to various growth factors and hormones [35]. DGKD was reported to interact with RACK1, a protein that we had previously demonstrated as an AR interacting protein that regulates AR phosphorylation and transcriptional activity [36], [37]. Thus, DGKD may contribute to AR regulation through RACK1. However, knockdown of DGKD did not have a significant effect on AR transcriptional activity. Previous research has shown that in the absence of DGKD, EGFR signaling is decreased because both expression and kinase activity are inhibited [38]. This effect on EGFR is a result of a decrease in a deubiquitinase, USP-8, and therefore increased ubiquitination and degradation of the EGFR [38]. Growth factor signaling is a known regulator of prostate cancer cell growth [29]. It is therefore possible that the growth effect that corresponds with DGKD knockdown is the result of altered receptor tyrosine kinase signaling.

ICK is a serine/threonine kinase containing a dual phosphorylation site found in mitogen-activating protein kinases whose activity is regulated by cell cycle-related kinase (CCRK) and human protein phosphatase 5 (PP5) [39]. ICK is related to male germ cell-associated protein kinase (MAK). MAK is an AR co-regulator that directly binds the AR in co-immunoprecipitation experiments and enhances AR-dependent transcription in a kinase-dependent manner [40]. Inhibition of MAK with either RNAi or a kinase-dead form decreased LNCaP cell growth. The effect of MAK knockdown on growth parallels our observations with ICK although we have not observed an effect of ICK on AR transcription suggesting an alternate mechanism of growth regulation. ICK can phosphorylate Scythe, an antiapoptotic protein and thus may regulate cell survival [39], [41].

CIT is a dual specificity protein kinase that is a putative RHO and RAC effector, which may play a role in cytokinesis [42]. CIT knockdown may disrupt cytokinesis and therefore cell growth. GALK2 is an N-acetylgalactosamine (GalNAc) kinase, which also has galactokinase activity at high galactose concentrations [43]. Regulating carbohydrate metabolism is necessary for cell growth and GalNAc is critical for O-linked glycosylation and the corresponding regulation of cell signaling [44]; GALK2 knockdown may disrupt these fundamental cellular processes. PSKH1 is an understudied kinase that may be a splicing factor compartment-associated serine kinase with a role in intranuclear non-snRNP splicing factor trafficking and pre-mRNA processing [45]. The absence of a major effect in the non-tumorigenic LHS prostate cells and MCF10A breast cells may be due to differences in the relative effectiveness of the knockdown and/or requirement for these kinases in these fundamental cellular processes. It is plausible that the requirement for CIT, GALK2, and PSKH1 varies depending upon the tumorigenic state since the mRNA levels of these kinases increase as prostate cancer progresses (**Figure S1**). Further study is required to determine the mechanism of growth regulation for these kinases in prostate cancer.

In this study we screened a panel of shRNAs targeting the human kinome against LNCaP prostate cancer cells grown in the presence and absence of androgen to identify the signaling pathways that regulate prostate cancer cell growth. We identified multiple shRNA clones against kinases that regulate both androgen-dependent and castration-resistant prostate cancer cell growth. This study further demonstrates the applicability of lentiviral based shRNA for conducting phenotypic screens to identify targets involved in a variety of biological processes, and establishes MAP3K11, DGKD, ICK, CIT, GALK2, and PSKH1 as regulators of prostate cancer cell growth.

## Materials and Methods

### Cell Culture

LNCaP and C4-2B cells (a gift from Dr. L. W. K. Chung) were grown in T-Medium (Invitrogen) with 5% Non-Heat Inactivated (NHI) serum (Gemini) as previously described [32], [46], [47]. For growth and RNA experiments, phenol free RPMI media (Invitrogen) with 5% Charcoal Stripped Serum (CSS) (Gemini) was used. Cells were stored in incubators at 37°C. LHS (a gift from Dr. William Hahn) and MCF10A (a gift from Dr. Sarah Parsons) cells were grown as previously described [25], [26], [32].

### RNAi Screen

MISSION®LentiExpress Human Kinase Panels, one panel in the presence of R1881 and one in the absence of R1881, were used in triplicate per condition. Controls included, in four wells per plate, lentivirus with shRNA against the androgen receptor (AR) (NM_000044). AR knockdown was stable under the experimental conditions of the assay (data not shown). Additional controls also included, in four wells per plate, the MISSION® pLKO.1-puro Control Transduction Particles and MISSION® Non-Target shRNA Control Transduction Particles. The LNCaP human prostate cancer cell line was reverse-transduced at 6,000 cells per well at multiplicities of infection (MOI) of approximately 1 in the presence of 8 µg/mL polybrene (hexadimethrine bromide), then incubated with virus for approximately 18 hours in a 37°C, 5% CO2 incubator. Medium was changed and contained either complete medium with 50 pM R1881 or complete medium prepared with charcoal-stripped FBS (csFBS). Forty-eight hours post-transduction, all panel plates were placed under 2 µg/mL puromycin selection, and at 96 hours post-transduction, medium was changed to fresh complete containing puromycin. On day seven post-transduction, medium was changed with resazurin solution added at 10% of the medium volume to all plates. Plates were incubated as described for three hours and fluorescence was read at an excitation of 560 nm and an emission of 590 nm. Data were analyzed by subtracting out background of complete medium plus resazurin, combining three biological replicates, and converting all transduced wells in both presence and absence of R1881 to a growth percentage as compared to MISSION® pLKO.1-puro Control wells, which were set at 100%.

### Growth Assays

10 MOI of shRNA virus or empty vector pLKO control virus was added to a 96 well plate previously coated with 1 µg/ml fibronectin. Puromycin +/− controls were also plated and did not contain virus. 6000 cells were then plated in RPMI media with 5% CSS with and without hormone (0.05 nM R1881 or ethanol). Cells were grown for 7 days under these conditions. On day 2, puromycin selection was added at a final concentration of 2 µg/ml to all wells except the puromycin (-) control. On day 4, media was changed on the plate, with selection and treatment as required. On day 7, CyQuant Assay (Invitrogen) was performed to determine cell number. A standard curve was plated with the appropriate cell line in T-Medium with 5% NHI serum with cell number varying from 25600 to 0. These cells were allowed to settle for 3–4 hours and after, all media was removed from the plate and replaced with 50 ml NF dye diluted 1:500 in 1X HBSS. The cells then incubated in the dark for 60 minutes at 37°C. Quantification was performed on a BioTek Synergy 2 plate reader.

### Immunoprecipitations and Western Blots

Immunoprecipitations, western blots, and quantitation were performed as previously described [32], [48].

### Statistics

For each cell line, two-way ANOVA was used to test for the effects of the knockdown and level of hormone. F-tests for the interaction were used to test whether the effect of the knockdown differed by hormone dose. When the interaction was not statistically significant, the effect of the knockdown was estimated by the main effect, averaging over levels of hormone. Growth measurements were transformed to the log scale to facilitate interpretation in terms of fold changes relative to control.

### RNA Isolation and qPCR

10 MOI of shRNA virus or empty vector pLKO control virus was added to a 12 well plate previously coated with 1 µg/ml fibronectin. Puromycin +/− controls were also plated and did not contain virus. 80,000 cells per well were plated in RPMI with 5% CSS. On day 2, puromycin selection was added at a final concentration of 2 µg/ml in all wells except the puromycin (-) control. On day 3, R1881 was added at final concentrations of 0.05 nM, 0.5 nM, 1 nM and ethanol vehicle alone. 2 hours and 24 hours after hormone addition, RNA was isolated using RNeasy Kit (QIAGEN, Chatsworth, CA) or preserved using RNALater (Ambion). RNA concentrations were determined using Ribogreen Assay (Molecular Probes, Inc., Eugene, OR). RNA was reverse transcribed using the iScript cDNA synthesis kit (Bio-Rad). Quantitative Real-Time PCR (qPCR) was done using a CFX96 (Bio-Rad). Target kinase mRNA was assessed using a TaqMan Probe (Applied Biosystems) and an IQ Supermix with FAM (Bio-Rad) and a 2-step PCR protocol with an extension and annealing temperature of 60°C. AR target genes and the housekeeping gene used IQ SYBR Green PCR master mix (Bio-Rad) or a master mix made with Taq polymerase (Roche), SYBR Green (Invitrogen). AQP3, FKBP51, GUS, Nkx3.1, PSA, PSMB6, SGK, STEAP4, and TMPRSS2 were run as described previously [32], [48]. CDC20, CDK1, and UBE2C primers and conditions were from [27]. DKK, FST, ORM1, and UGT2B were run on a 3-step protocol with an annealing and extension temperature of 60°C (*DKK* forward: 5′–CCTTGGATGGGTATTCCAGA–3′; *DKK* reverse: 5′–CAGTCTGATGACCGGAGACA–3; *FST* forward: 5′– TGTGCCCTGACAGTAAGTCG–3′; *FST* reverse: 5′– CCGAAATGGAGTTGCAAGAT–3; *ORM1* forward: 5′– GGGTCATTTCCACCACCTCAAACA–3′; *ORM1* reverse: 5′– GGAGAAAGGCCTTACAGTAGTCTC–3; *UGT2B* forward: 5′– ATGGGAATAAACCAGATGCC–3′; *UGT2B* reverse: 5′– GATCCCATGGTAGATTGCCT–3).

## Supporting Information

Figure S1 **Oncomine analysis.** We examined gene array data from Oncomine for changes in kinase expression over prostate cancer disease progression. Shown are box plots from two independent gene array studies for six kinases that increase in expression in either primary prostate cancer as compared to normal prostate or increased in metastatic prostate cancer when compared to primary disease. In each plot, 1 is the more benign and 2 is the more advanced stage of disease.
(TIF)

Figure S2 **Kinase target knockdown across hormone dose.** Targeted shRNAs knockdown kinase transcript levels in LNCaP (A) and C4-2B (B) cells. qPCR measured transcript levels of six kinases after the transduction of two shRNAs per kinase and pLKO empty vector control. RNA was isolated at 24 hours after the addition of R1881 at varying concentrations (vehicle, 0.05, 0.5, and 1 nM). The transcript levels were compared to pLKO (-) and normalized to the housekeeping gene, PSMB6. Error bars represent standard error of the mean.
(TIF)
