# Supplementary figures and images for: Identification of Kinases Regulating Prostate Cancer Cell Growth Using an RNAi Phenotypic Screen
Source: PLoS One. 2012 Jun 27;7(6):e38950. doi: 10.1371/journal.pone.0038950 (PMC3384611; doi:10.1371/journal.pone.0038950)

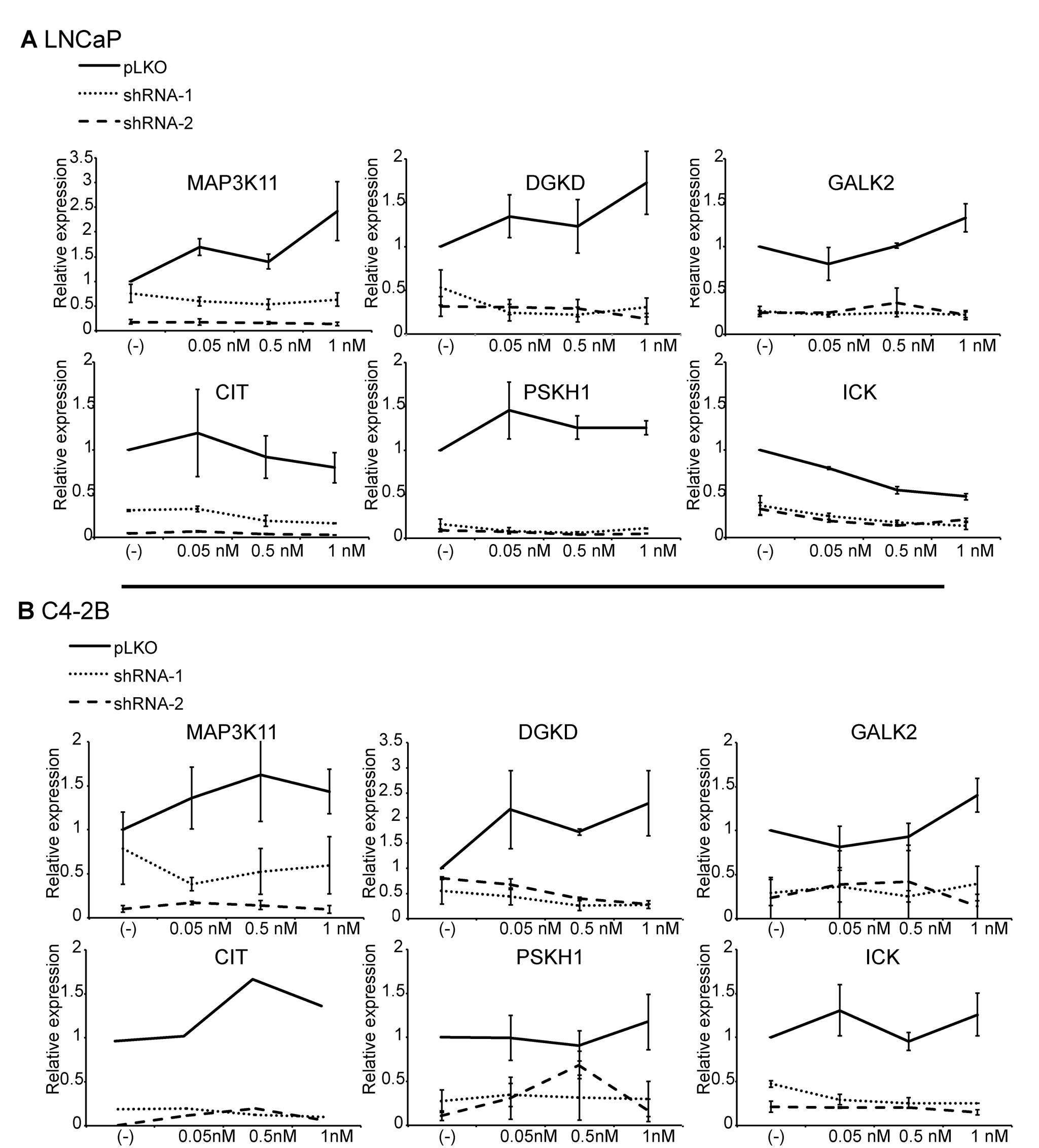

Supplement: Figure S1 — Oncomine analysis. We examined gene array data from Oncomine for changes in kinase expression over prostate cancer disease progression. Shown are box plots from two independent gene array studies for six kinases that increase in expression in either primary prostate cancer as compared to normal prostate or increased in metastatic prostate cancer when compared to primary disease. In each plot, 1 is the more benign and 2 is the more advanced stage of disease. (TIF) [file pone.0038950.s001.tif]

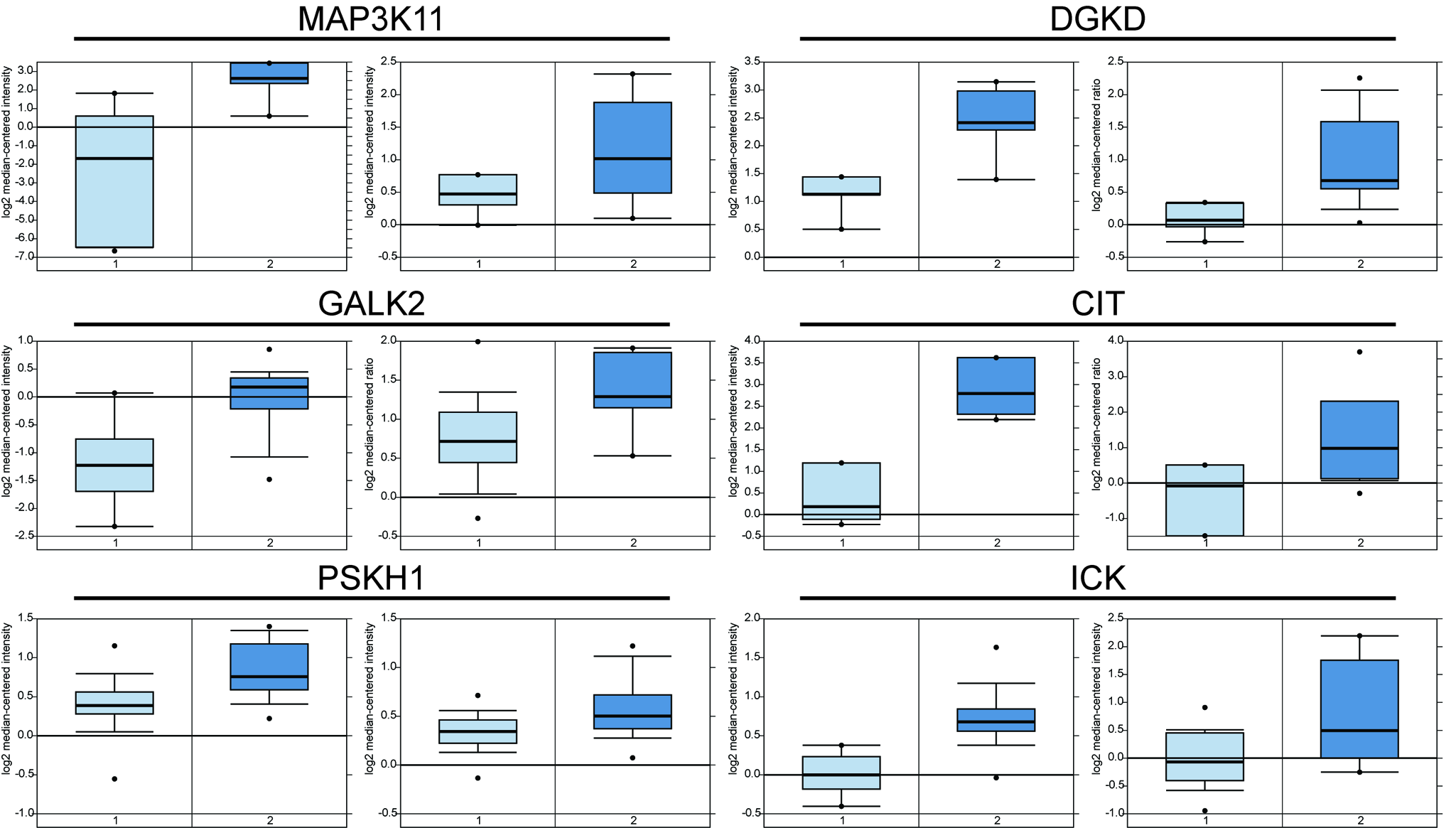

Supplement: Figure S2 — Kinase target knockdown across hormone dose. Targeted shRNAs knockdown kinase transcript levels in LNCaP (A) and C4-2B (B) cells. qPCR measured transcript levels of six kinases after the transduction of two shRNAs per kinase and pLKO empty vector control. RNA was isolated at 24 hours after the addition of R1881 at varying concentrations (vehicle, 0.05, 0.5, and 1 nM). The transcript levels were compared to pLKO (-) and normalized to the housekeeping gene, PSMB6. Error bars represent standard error of the mean. (TIF) [file pone.0038950.s002.tif]
